# Study protocol for evaluating automation of systematic review processes with EPPI-Reviewer and Copilot 365 in updating the cataract evidence gap map

**DOI:** 10.1186/s13643-026-03101-4

**Published:** 2026-02-17

**Authors:** Bhavisha Virendrakumar, Hugh Sharma Waddington, Pauline Scheelbeek, Emma Jolley, Elena Schmidt

**Affiliations:** 1 London School of Hygiene and Tropical Medicine, Keppel St, London, WC1E 7HT UK; 2 Sightsavers, 35 Perrymount Road, Haywards Heath, RH16 3BW UK

**Keywords:** Artificial intelligence, Systematic review, Large language models, Protocol, Accuracy, Priority screening, Automation, Data extraction, Critical appraisal, Copilot 365

## Abstract

**Background:**

The process of developing and updating an evidence gap map (EGM) is based on the principles of systematic reviews and requires extensive time and financial resources. Artificial intelligence (AI) tools, like prioritisation screening (PS), integrated into programmes such as EPPI-Reviewer (ER) and Copilot 365, can potentially mimic human performance in systematic review processes. ER is a subscription-based web application employed by systematic review groups, while Copilot 365, integrated into Microsoft 365, offers real-time assistance. Although ER shows promise in speeding up screening, the optimal threshold for accuracy remains unclear. Additionally, there is no evidence on the effectiveness of any version of Copilot in systematic review and EGM processes.

**Objectives:**

Assess the accuracy and efficiency of Copilot 365 and PS integrated into ER at different stages of an EGM update, comparing it to human performance.

**Methods:**

We will conduct both manual and automated screening of references, full-text screening, data extraction, and critical appraisal. Two reviewers will independently screen studies for inclusion, extract data, and appraise included studies, resolving conflicts through discussion. We will assess the accuracy and efficiency of Copilot 365 and ER at different EGM update stages, comparing them to human performance. To evaluate the PS accuracy, we will use 20% and 40% manual screening thresholds, calculating the proportion of relevant references prioritised by PS and the total relevant citations missed. We will compare Copilot 365’s full-text screening accuracy to reviewers’ decisions and assess consistency using Cohen’s Kappa. For automated data extraction and appraisal, we will manually inspect 20% of Copilot 365’s outputs, comparing them to reviewers’ results, measuring consistency with Cohen’s Kappa, and evaluating time savings by comparing the time taken for manual extraction versus using Copilot 365.

**Discussion:**

This study will offer insights into ER’s accuracy in screening small samples of citations and potentially guide future applications in this context. Additionally, by evaluating Copilot 365, which shares similar features with other AI tools, we will gain a broader understanding of its applicability and limitations in evidence synthesis, making the results relevant to other AI applications in this field.

**Systematic review registration:**

Registered at Open Science Framework: https://doi.org/10.17605/OSF.IO/49BX8.

**Supplementary Information:**

The online version contains supplementary material available at 10.1186/s13643-026-03101-4.

## Background

An Evidence gap map (EGM) is a tool for presenting the state of the available evidence on a topic and highlighting gaps for future research in a user-friendly format [1]. Sightsavers’ EGMs summarise, appraise, and present evidence from systematic or literature reviews relevant to low- and middle-income countries across five eye health areas: cataract, glaucoma, diabetic retinopathy, refractive error, and trachoma [2, 3]. EGMs and their associated outputs have been demonstrated to be valuable tools for decision-making across different fields [4, 5]. The process of constructing the EGMs is based on the principles of systematic reviews and requires extensive time, human, and financial resources. It involves a systematic search of the literature, and subsequent screening of identified literature, extraction, and critical appraisal of reviews by two (or more) reviewers independently [1, 3]. In addition, a summary of each review is thereafter produced including details relating to reviews’ objectives, methods, findings, applicability and generalisability of findings, and critical appraisal assessment.

To ensure that policymakers, clinicians, and researchers benefit from the most up-to-date evidence, each EGM is updated every 3 to 4 years. The update process follows the same approach as the original [3] and, depending on the availability of resources, full updates of EGMs can take between 6 and 10 months to complete. Therefore, by the time an EGM is updated, its content may not be representative of the most current evidence, particularly in a rapidly growing research field [6].

### Cataract EGM

The cataract EGM, updated in December 2021, includes 98 systematic or literature reviews across five sectors: biomedical research, service delivery, and impact/economic evaluations. Key insights from the map highlight a significant gap in reviews addressing health systems, which is crucial as we strive towards universal health coverage and health systems strengthening. It also underscores the need for reviewers to maintain high methodological standards and transparency, given the critical role of synthesis work in decision-making. Since its inception in 2015, the number of reviews in the cataract map increased by approximately 88%, reflecting a growing interest in cataract research and its public health implications. Over time, the literature on cataracts is anticipated to expand due to the aging population and the increasing prevalence of this condition [7, 8]. Additionally, innovations in medical technology and surgical techniques are expected to drive more studies aimed at enhancing cataract diagnosis and treatment outcomes [9].

Over the past few years, artificial intelligence (AI) tools have gained attention, particularly for their potential in streamlining systematic review tasks, including those employed for developing or updating EGMs [10, 11]. AI tools have the potential to speed up the process of developing and updating EGMs, with the same or fewer resources, and thus improve the timeliness and completeness of evidence available to policy makers and other consumers [12].

A glossary has been developed to define AI-related terms and metrics mentioned in this protocol (see Additional file 1).

### What is artificial intelligence?

AI enables computers and machines to mimic or simulate human intelligence and problem-solving capabilities [13]. Machine learning (ML), a subset within AI, focuses on the use of various ML algorithms that derive knowledge from data to predict outcomes. ML algorithms include both supervised (where data is labelled by humans) and unsupervised learning (where the algorithm finds patterns in unlabelled data). Deep learning (DL) algorithms, a subset of ML, allow for unsupervised learning, where the algorithm automates the extraction of features from large, unstructured, or unlabelled data sets. Given that it operates without human intervention, DL enables ML at scale [13]. Examples of ML include chatbots and virtual assistants (e.g. Alexa) and recommendation systems (e.g. Netflix/Amazon recommendations). DL examples include generative AI (e.g. ChatGPT–generative pre-trained transformer) [13] and self-driving cars [14].

Natural language processing (NLP), another commonly used term, is a branch of computer science and AI that leverages ML to help computers comprehend and interact with human language. Research in NLP has paved the way to the era of generative AI, enhancing the communication abilities of large language models (LLMs) and enabling image generation models to comprehend requests. NLP use cases include grammar correction—when NLP functions are trained to identify grammar errors and suggest corrections—and machine translation—effective translation portraying the meaning and tone of the source language, ensuring the translated text retains the same meaning and desired impact in the target language [15].

### Automation in systematic review processes

In the evidence synthesis world, AI has shown the ability to handle tasks that traditionally demand human intelligence or intervention [11]. With evolving algorithms, AI has the potential to quickly mimic human performance in systematic review processes [10, 16], including searching and importing documents, screening, data extraction, and presentation of review outputs. At the time of writing this protocol, the digital evidence synthesis tool (DEST) identified 111 automated tools in the context of climate and health [17, 18], and the systematic review toolbox identified 225 software tools and 78 guidance documents applicable to systematic reviews [19].

Previous research, comparing different AI tools, found the following supporting the greatest number of features for systematic reviews: DistillerSR (Evidence partners), EPPI-Reviewer (EPPI-Centre), SWIFT-Active Screener (Science), and Covidence (Cochrane) [20, 21]. Automated approaches have been shown to save time and other resources when selecting relevant documents [12]. Text mining has been shown to support managing the screening of large amounts of literature [22], and as an alternative to the traditional double screening approach [16, 23]. Evidence further suggests the potential of ML classification algorithms (known as classifiers) in reducing the manual study identification workload at screening stages [24]. Although there has been an increase in reviews automating data extraction and critical appraisal, these processes are still at the piloting stage and require a great deal of human input [25, 26].

LLMs have promising capabilities for automating systematic review processes including data extraction and critical appraisal, given their ability to generate context-specific information in a narrative format [17]. Evidence exploring ChatGPT, a generative AI, has shown it to be a valuable tool across different areas, including scientific writing such as abstracts, background, and generating summaries of research articles [27]. A study automating and streamlining systematic review steps using ChatGPT found promising findings in relation to screening and categorisation of relevant studies—ultimately reducing human efforts and time. However, study authors found limitations in its suitability for extracting data from included studies [27]. Additional limitations include its knowledge cut-off date of June 2024 (at the time of writing this protocol) when using the free version of ChatGPT, indicating no access to any information on publications published after that date. This further implies that its responses are based on patterns it observed in the training data, and it does not actively search the internet for real-time information [27]. Despite the popularity and potential of ChatGPT, evidence suggests the need for caution when using it. One study, exploring ChatGPT capabilities in providing safety-related advice, found significant risks when used as a source of information and advice, with AI providing incorrect or potentially harmful information [28].

Assessing and comparing the features of each tool can assist the review community in deciding which tool to use. However, the continuous evolution and updating of AI tools present a challenge for researchers in selecting the most suitable tool that also addresses ethical, security, and other considerations. Current research studies employing AI for evidence synthesis highlight inconsistencies in reporting standards across disciplines [29], a lack of universally agreed-upon reporting standards for evidence synthesis methods and the use of digital tools, a scarcity of studies rigorously evaluating the reliability of these tools, and a lack of formal evaluations on how tools with different functionalities are utilised across the various stages of the review [17]. The systematic review toolbox suggests that many software tools and guidance documents could be relevant to multiple review types, although reviews of reviews and qualitative reviews may currently be less well served. Additionally, most guidance documents focus on methods for critical appraisal followed by reporting guidelines, with fewer publications addressing other aspects of the review production process [19].

While AI has the potential to automate tasks, studies highlight ethical issues such as bias from self-learning or ML algorithms, the use of inappropriate training data, lack of transparency in complex ML algorithms and LLMs, unrepresentative datasets leading to equality concerns, and cybersecurity risks [30]. Furthermore, the “black box” nature of AI tools, where decisions are made without clear explanations, undermines the principles of systematic reviews’ replicability and may pose concerns about the validity and trust in the automated review process [29–31].

### Automation of EGMs

Automation can significantly advance and mainstream EGMs processes, helping bridge the gap between the availability of evidence and the needs of decision-makers [17]. A study suggests that incorporating the Bidirectional Encoder Representations from Transformers (BERT) language model, a ML framework for NLP [32]. Alongside human reviewers, can improve efficiency. In the design of three EGMs integrating BERT-based AI agent into the human team, a study found reduced human screening by approximately 65% compared to traditional methods and by 16% when using a support vector machine (SVM)-based AI agent for identifying 85% of all relevant documents. The same study reported higher classification accuracy (proportion of correct classifications) of documents when using BERT model compared to SVM model (0.82 vs. 0.7) [4]. Supervised machine learning, which involves training models by human-provided examples, has shown to aid in the selection and categorisation of scientific publications for a climate-related evidence map [17, 33]. Unsupervised machine learning, employed in an evidence map for climate impact, helped in identifying patterns and develop an initial understanding of relationships within large, unfamiliar sets of scientific publications [17, 34]. Despite the potential benefits of automation of EGMs, current evidence is limited to tasks such as literature searching, selection and deduplication. Moreover, appropriate quality standards for automated EGMs in terms of precision and recall remain unclear [17].

## Tools of interest: EPPI-Reviewer and Microsoft Copilot 365

ER, a web-based tool developed at University College London, is popular among systematic review groups. Although PS, integrated into ER, has the potential to reduce screening burden and review workload, few studies have evaluated its accuracy in updating EGMs.

Copilot 365 was selected based on its data and security compliance and its capabilities in supporting organisations to understand, summarise, predict, and generate content from vast data sets, making it a suitable contender for evaluation in the context of EGMs. Additionally, to our knowledge, no studies have assessed Copilot 365.

### EPPI-Reviewer

EPPI-Reviewer (ER) is a widely used tool that integrates ML software at different stages of a systematic review. It is recommended by both the Cochrane Collaboration and the International Initiative for Impact Evaluations (3ie) [35]. As a web-based platform, ER facilitates real-time collaboration between institutions and reviewers. Reviewers can screen documents for eligibility by examining titles, abstracts, and full texts. The platform also includes pre-built tools for data extraction and assessing risk of bias, along with options to create customised templates for data extraction and synthesis. Additionally, ER supports qualitative synthesis of studies using the ‘line-by-line’ coding approach for PDF documents, which can be combined with free text reports in customised formats. The system further provides access to statistical analysis tools, including those for network meta-analysis, and features a relational data extraction system [35].

The integrated automated tools in ER include features such as deduplication, automatic clustering of studies, classifiers, and priority screening (PS). The PS tool requires a human reviewer to identify at least six includes and six excludes to allow the algorithm to start learning. As the algorithm processes this information, the PS rearranges the list of unscreened references, placing those most likely to be included at the top and those unlikely to be included at the bottom. References that the algorithm is unsure about are placed randomly in the list [36]. On the other hand, PS does not provide clear thresholds or cut-off points for included, excluded, or unsure references. The PS process is dynamic, meaning the order of references constantly adjusts as reviewers assess their eligibility. Therefore, the more references are screened manually, the more accurate the algorithm becomes, leading to better placement of included and excluded references on the list. Other tools integrated into ER include Open Alex, which supports automated review updates, and ChatGPT (under development and evaluation) to assist with automated data extraction [35].

Research indicates that implementing ER in creating an EGM can cut overall workload by almost 90% [12, 16]. However, specific contributions of automated tools remain unclear. Studies assessing the PS function within ER for different reviews has demonstrated potential for reductions in screening burden resulting in savings in the number of title and abstracts that needed to be screened. Ultimately, saving time and effort. The water, sanitation and hygiene EGM study estimated saving 90% of screening costs using PS with a stopping criterion of 100 successive negative records and random screening of 100 citations of the remaining studies to ensure that no studies were missed [12]. Another study demonstrated that different thresholds of manual screening were required for ER to achieve 100% sensitivity (ability to identify all relevant studies) in larger reports, ensuring all relevant articles were identified. Specifically, the thresholds were 39.9%, 71%, and 91%. For smaller reports, the study found ER provided benefits in reducing the screening burden, but the gains were more modest compared to larger reports due to the smaller size of training sets. The thresholds for achieving 100% sensitivity for various smaller reports ranged between 86 and 97% [37]. However, this study employed the star rating systems previously available at ER. Furthermore, it remains unclear the optimum threshold for resulting in accurate prioritisation screening using ER [38].

### Microsoft 365 (Copilot 365)

Copilot for Microsoft 365 (Copilot 365) is an AI tool that integrates LLMs, a form of AI algorithm that employs deep learning techniques and vast data sets to understand, summarise, predict, and generate content. These LLMs, like GPT-4, are pre-trained models specifically designed to excel in these tasks, similar to ChatGPT. Copilot 365 is integrated within Microsoft Graph and Microsoft applications including Word, Excel, Outlook, Teams, PowerPoint, and OneDrive [39, 40]. By integrating with OneDrive, Copilot 365 can generate responses based on personal data sources, such as documents stored on SharePoint and OneDrive. This enables the tool to summarise and extract relevant data from a particular file or group of files into different formats, thereby unlocking relevant information and generating insights [40, 41]. Additionally, Copilot 365 has internet access via the Bing search engine, allowing it to draw and reference information as needed. Furthermore, this tool is designed with a strong focus on data protection. It adheres to privacy, security, and compliance commitments, including the General Data Protection Regulation, offering a higher level of protection compared to ChatGPT. Unlike ChatGPT, Copilot 365’s language model is not trained on user-provider prompts, responses, or data, ensuring enhanced privacy [39, 41, 42].

Given Copilot 365’s potential and its strong data protection policy, non-governmental organisations have begun exploring various use cases and testing different versions of Microsoft Copilot [42]. For instance, Eric Couper from the International Youth Foundation highlighted the exploration of Core Copilot, Copilot 365, and Copilot Studio, advocating for their adoption due to their unique functionalities tailored to diverse needs. At Mercy Corps, the director of monitoring, evaluation, and learning technologies identified several use cases for the free version of Copilot, including drafting narrative reports, creating slide decks from reports, conducting deep analyses of qualitative data, and reviewing documents. However, preliminary findings from Mercy Corps are inconclusive, highlighting the need for further testing to determine its cost-effectiveness [42]. To date, there is no evidence exploring the effectiveness of any version of Copilot in systematic review and EGM processes.

### Automation of cataract EGM: potential challenges

The body of literature on cataracts is growing due to their increasing prevalence and advancements in medical technology [43]. Existing research synthesising evidence on AI in cataract studies primarily focuses on its application for surgery and management [44, 45]. To the best of our knowledge, there are no studies automating cataract-related systematic reviews or EGMs. While AI algorithms can alleviate the screening burden for reviewers, we may encounter challenges during the cataract EGM update when employing automated approaches. Reviews on the cataract EGM cover complex subjects (i.e. epidemiology, treatment), study designs, population, interventions and outcomes, which require broad search strategies to address wide-ranging research questions. This complexity makes manual screening of reviews for inclusion and exclusion challenging. Consequently, AI algorithms may also struggle to grasp the reviewers’ thought process regarding inclusion and exclusion criteria [16, 46]. Moreover, the variability of information, terminology, and structure presented in abstracts vary can make it difficult for AI algorithms to accurately assess relevance [46]. Imbalanced datasets, where irrelevant studies outnumber relevant ones, as seen in typical EGMs or systematic review, may pose additional challenges for AI algorithms during the title and abstract screening stage [16]. If reviewers do not manage to sift through a large a number of irrelevant studies during the AI algorithm’s training, the algorithm may overlook relevant studies that should be included [47, 48].

Furthermore, research indicates that for a screening tool like ER to perform accurately during the title and abstract screening stage, the sample size must be sufficiently large (around 2000 and above) for the algorithm to learn effectively [37]. This poses a challenge for this study, as the search is expected to yield a small sample size (approximately 300 citations) due to the restriction to reviews published between 2021 and 2024. This limited sample size may result in a constrained training dataset for the algorithm to learn from LLMs, such as Copilot 365, offer promising capabilities to support data extraction and critical appraisal processes for the cataract EGM update [49]. However, given the diversity of reviews eligible for inclusion in the cataract EGM, Copilot 365 may face several challenges:

- Consistently extract data and critically appraise across different domains or study designs [50].
- Accurately interpreting and extracting relevant information from complex and nuanced texts [50]
- Understanding the context in which certain data are presented, such as distinguishing between primary and secondary outcomes and exploratory analyses [50].

These challenges can lead Copilot 365 to produce errors or “hallucinations” when automating such activities. Mistakes in data extraction and appraisal assessment stages can propagate through the EGM update process, potentially leading to inaccurate conclusions [50]. Despite these challenges, previous research and advancements in AI indicate that the capabilities of LLMs in this area are promising and continually improving [49].

### Study objective

This research aims to evaluate the accuracy and efficiency of Copilot 365 and PS integrated into ER at different stages of an EGM update, comparing it to human performance. Specially, this study will involve updating the cataract EGM [43] as a case study. Ultimately, this assessment will help us understand the benefits and drawbacks of these AI tools, inform new approaches for updating Sightsavers’ EGMs, guide the development of trustworthiness, and pave the way for review teams to adopt these AI tools more efficiently.

### Research questions

The study will address the following questions, which are described in more detail below:

1. What is the accuracy of the PS algorithm in prioritising relevant references at 20% and 40% manual screening thresholds?
2. What is the accuracy and consistency of Copilot 365 when screening the full text of reviews for inclusion and exclusion compared to human performance?
3. What is the accuracy and efficiency of Copilot 365 in extracting data and critically appraising reviews compared to human reviewers?
4. What is the completeness of data extraction and appraisal performed by Copilot in comparison to manual processes?

## Methods

We prepared this protocol following the Preferred Items for Systematic Review and Meta-Analysis Protocols (PRISMA-P) [51].

Before drafting this protocol, the study lead (BV) explored and employed PS during the title and abstract screening stage for updating an EGM to understand the tools’ functionalities. In addition, Copilot 365 was explored to learn its features and prompts to be employed during the study.

### Ethics approval

Ethics approval was not sought as this study will not involve human participants and collecting identifiable data.

### Study registration

The protocol is registered at Open Science Framework: 10.17605/OSF.IO/49BX8.

### Test methods

In this study, we will assess the accuracy of PS, integrated into ER, at 20% and 40% manual screening thresholds at the title and abstract screening stage. Copilot 365 will be assessed in terms of accuracy, efficiency, and/or consistency at the full text screening stage, data extraction, and critical appraisal of reviews eligible for inclusion in the cataract EGM. See Table 1 for definitions of performance metrics used in this study protocol.

**Table 1:** Definitions of performance measures

Measure	Definition
Accuracy	The degree to which the automated system, Copilot 365, correctly identifies relevant studies, extracts, and appraises relevant data compared to human reviewers
Efficiency	Refers to how effectively AI tools perform tasks compared to human performance, including accuracy
Consistency (inter-reliability)	The degree to which different reviewers (human or AI) agree on the inclusion or exclusion of studies, on data extraction and critical appraisal assessments
Heuristic cut-off point	Reviewers stop screening citations when a given number of irrelevant articles are seen in a row
Time savings	The reduction in time required to perform data extraction and critical appraisal tasks when using the automated system, Copilot 365, compared to manual efforts by human reviewers

The study lead will collaborate with two team members to compare the performance of the selected AI tools with the gold standard approach, where two human reviewers independently screen reviews for inclusion, extract data, and appraise eligible reviews. One team member will have extensive cataract-related expertise in the context of low- and middle-income countries, while another will be experienced in evidence synthesis methods throughout all stages of EGM development. The detailed contribution of each reviewer is outlined below.

### Title and abstract screening and selection process

In this study, we will assess the proportion of relevant references (defined as likely includes) identified by PS at 20% and 40% thresholds relative to the total number of relevant references identified manually across the entire sample, based on title and abstracts only. In addition, we will determine the total number of relevant references not identified by PS at both thresholds.

Manual screening of the entire sample will be conducted by two reviewers independently in blind mode using Rayyan. Reviewers will categorise each reference based on title and abstract as ‘include’, ‘exclude’ or ‘maybe’. Reviewers will provide reasons behind each exclusion (e.g. wrong study design, wrong population). One reviewer, with a clinical background and extensive experience in the field of cataracts in the context of low- and middle-income settings, will assess references’ eligibility based on its relevance. The second reviewer, with expertise in evidence synthesis methodology, will ensure that references are included based on methods—systematic and literature reviews. Once completed, we will run a report to compare decisions made by each reviewer. Conflicted inclusion and exclusion decisions between reviewers and those labelled as ‘maybe’ will be resolved through discussion. In case of disagreement, a third reviewer will be consulted. Following consensus, the reviewer whose decision differs from the agreed result will update their submitted decision on the platform to align with the consensus. After the manual screening is complete, we will initiate reference screening with ER using the same references that were manually reviewed.

Prior to beginning the eligibility assessment of references for inclusion or exclusion using ER, PS will be enabled to ensure the algorithm learns through reviewers’ decisions. As it learns, PS will iteratively and automatically reorganise the reference list, placing those most likely for inclusion at the top, less likely for inclusion at the bottom, and ‘unsure’ references randomly in the list.

**Fig. 1:**
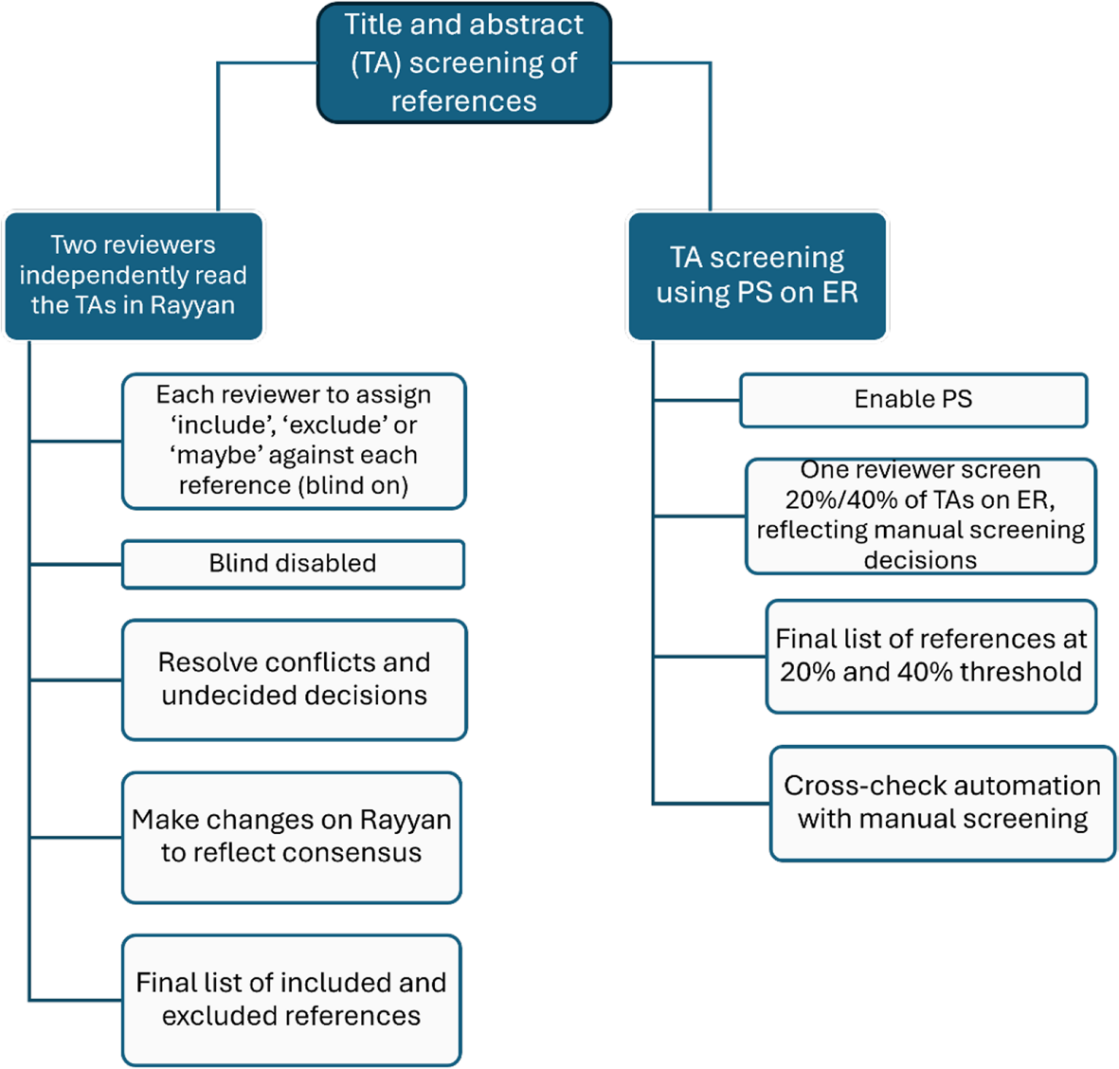
Manual and automated title and abstract screening methods

The assessment process will involve one reviewer manually assessing 20% and 40% of references of the total sample size imported in the ER software. The reviewer will allocate the following codes against each reference: ‘include based on title and abstract’ or ‘exclude’. Include and exclude decisions will reflect the decisions made at the manual screening stage conducted by two reviewers. For each exclude, the reviewer will include reasons for exclusion. Once the 20%/40% of references have been assessed, the PS functionality will be disabled. The ER developers will be approached at the two different thresholds for the final reorganised list of references, which will be used to compare with the complete list of manually screened references (Fig. 1).

Since PS does not specify exact cut off points for includes and excludes but instead ranks likely includes higher on the list and likely excludes lower, we will employ a heuristic cut-off point [38]. This approach will allow the reviewer to determine likely excludes when a given number of irrelevant studies are seen in a row [38, 52, 53]. Based on prior research, we will adapt a cut-off of 25 consecutive irrelevant citations at each threshold, reflecting the anticipated small sample size of citations needed to be screened (Table 2) [38].

**Table 2:** Criteria for PS include and exclude cut-off points

Criteria	Cut-off points
References placed at the top of the list (likely includes)	Top of the list until likely exclude references are identified
References placed closer the bottom of the list (likely excludes)	Starting point defined when at least 25 references are identified as excludes by the reviewers

### Full-text screening and selection process of reviews

**Fig. 2:**
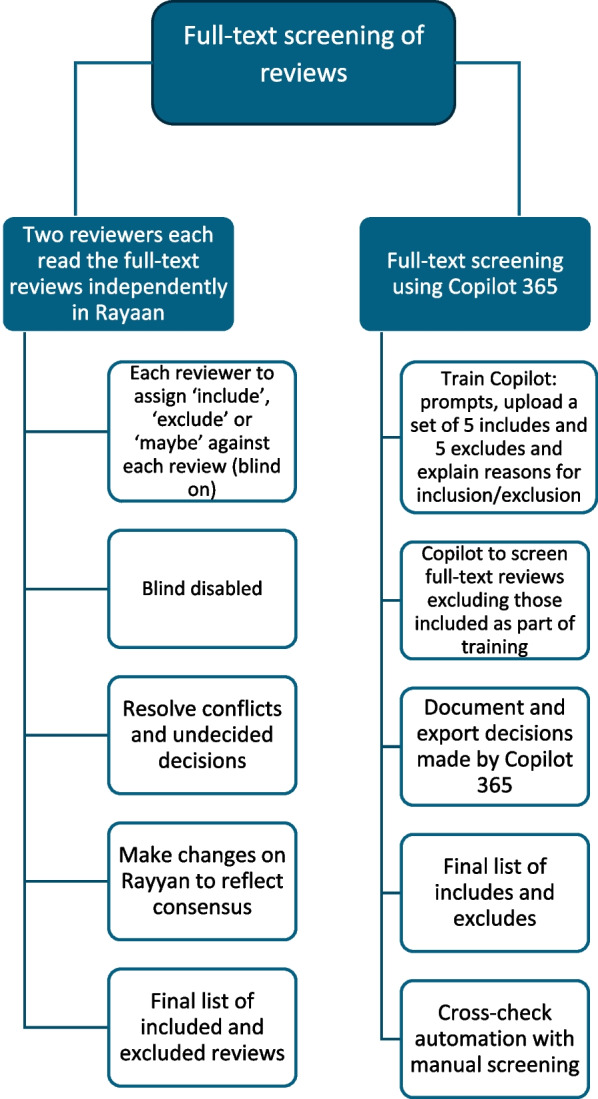
Full-text screening using manual versus automated techniques

To evaluate the automation of screening reviews based on full text, we will employ Copilot 365. We will assess the accuracy and consistency of Copilot 365 by comparing its inclusion/exclusion decisions with those made by human reviewers. The process involves first gathering the full texts of reviews that were identified for potential inclusion during the title and abstract screening stage. Following this, using Rayaan, two reviewers will independently and manually review each full-text document to determine its eligibility according to the criteria outlined in Table 3. The evidence synthesis methodologist involved in the title and abstract screening will also participate in reviewing the complete texts. The second reviewer will be an expert in conducting systematic reviews (Fig. 2).

**Table 3:** Criteria for inclusion and exclusion

Inclusion criteria	Exclusion criteria
Reviews containing a combination of evidence from high-, medium- and low-income settings; low-income countries; medium-income countries; or a combination of low- and medium-income countries. For clarity, we will use the World Bank’s income classifications [54]	Reviews containing evidence solely from high-income countries [54]
^a^Systematic reviews of ^b^effectiveness trials (effectiveness trials measure the beneficial impact under "real world" settings)	Systematic reviews of ^c^efficacy trials (efficacy trials assess whether an intervention yields the expected result under ideal or controlled conditions
Systematic reviews published from 1993 onwards	Systematic reviews published before 1993
^d^Narrative reviews that describe the methods used for data collection and synthesis	Narrative reviews lacking descriptions of the methods used for data collection and synthesis
Evidence appropriate for answering the question posed in the review	Evidence inappropriate for answering the question posed in the review (e.g., using non-causal evidence to answer a causal query)”

^a^Systematic reviews – gather evidence that meets specific criteria to answer a research question. They aim to reduce bias by following clear, pre-planned methods outlined in a protocol [55]

^b^Effectiveness trials - measure the degree of beneficial effect under “real world” clinical settings [56, 57]

^c^Efficacy whether an intervention produces the expected results under ideal circumstances [57]

^d^Narrative reviews - a review of the literature that provides comprehensive, descriptive summary of existing research on a topic [58]

Reviewers will assign each review as either ‘include’ or ‘exclude’, providing reasons for exclusion (e.g. efficacy trial). During the screening process, blinding will be enabled, preventing reviewers from seeing the decisions of their counterparts, only allowing them to view their own choices. Once the sifting phase is complete, blinding will be lifted, and decision outcome comparison reports will be generated. Any conflicts will be resolved through discussion, and if needed, a third opinion will be sought for disputed cases. Upon reaching consensus, adjustments to decision outcomes will be recorded in Rayaan to reflect the final agreed-upon decisions. This list, containing detailed decision outcomes, will be exported as an Excel file for comparison with Copilot 365’s automated screening.

The screening automation process will include firstly a training phase, led by the evidence synthesis methodologist, where the AI learns from manual decisions, progressively fine-tuning its algorithms to improve future screening accuracy. During the training phase, the reviewer will create the following prompt for Copilot 365:

“I am updating the cataract evidence gap map to include the most up-to-date systematic and literature reviews. I am at the stage of screening full texts of potentially eligible reviews for inclusion in the evidence gap map. The criteria to be used to determine inclusion and exclusion eligibility include:”

**Table:** 

Inclusion criteria	Exclusion criteria
Reviews containing a combination of evidence from high-, medium- and low-income settings; low-income countries; medium-income countries; or a combination of low- and medium-income countries. For clarity, we will use the World Bank’s income classifications	Reviews containing evidence solely from high-income countries
Systematic reviews of effectiveness trials (effectiveness trials measure the beneficial impact under "real world" settings)	Systematic reviews of efficacy trials (efficacy trials assess whether an intervention yields the expected result under ideal or controlled conditions
Systematic reviews published from 1993 onwards	Systematic reviews published before 1993
Narrative reviews that describe the methods used for data collection and synthesis	Narrative reviews lacking descriptions of the methods used for data collection and synthesis
Evidence appropriate for answering the question posed in the review	Evidence inappropriate for answering the question posed in the review (e.g., using non-causal evidence to answer a causal query)”

To train Copilot 365 to assist with the screening process, the reviewer will.

1. Provide Copilot 365 with a set of full-text reviews that have already been screened, including 5 of each included and excluded reviews.
2. For each excluded/included review, the reviewer will include an explanation of why it was excluded/included based on the criteria above.

Following training, the same reviewer will upload the remaining full text reviews individually and prompt Copilot 365 as follows:

“Should the attached review, named X, be included or excluded in the cataract evidence gap map based on the inclusion and exclusion criteria above? Provide your reasons for inclusion or exclusion.”

**Fig. 3:**
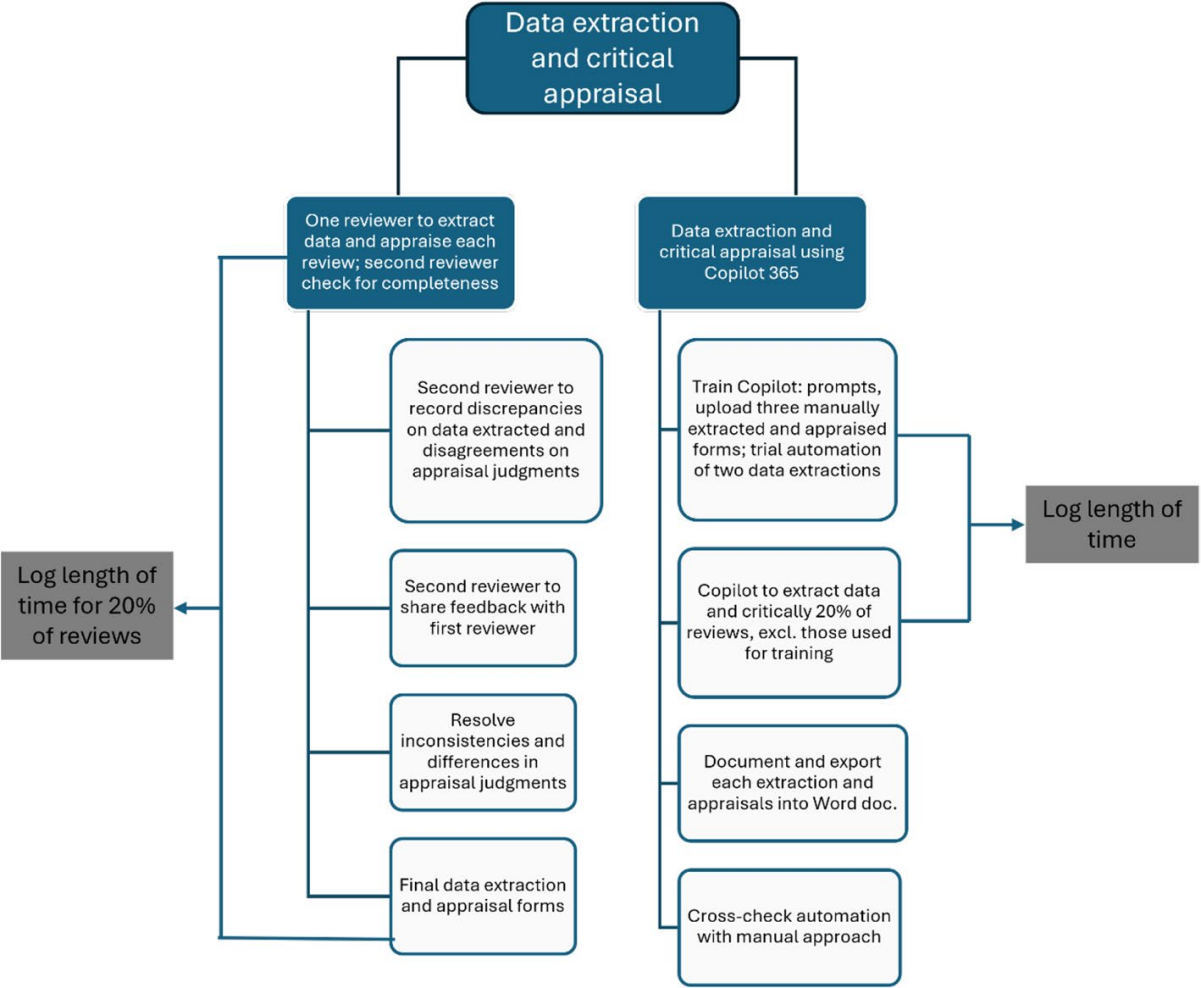
Manual and automated processes for data extraction and critical appraisal

The inclusion decisions made by Copilot 365 will be cross-checked with manual screening results to ensure consistency. Automated screening outcomes and reasons will be recorded to facilitate a detailed analysis, highlighting areas where the AI tool’s decisions align or diverge from human reviewers (Fig. 3).

### Data extraction and critical appraisal

This study will compare data extraction and critical appraisal automation using Copilot 365 with human performance. Two reviewers will manually extract data and appraise reviews at the full-text screening stage using the SURE checklist (Additional file 2). One reviewer will complete data extraction and appraisal, while the second reviewer will verify accuracy and completeness, checking for agreement. Discrepancies will be noted and discussed to resolve differences. Consensus forms will then be used to validate Copilot 365’s automated data extraction and appraisal decisions.

Automating both tasks will require training Copilot 365 by uploading three manually extracted and critically appraised forms alongside their corresponding full text reviews. The reviewer, involved in all stages of this study, will input the following prompt:

“I am updating the Sightsavers cataract evidence gap map to include more recent systematic or literature reviews. I am currently extracting data and critically appraising each review using the SURE checklist. The data extraction and appraisal are based on the full text of the review. Here is an example attached: the PDF is the full text of a review, and the Word document contains the SURE checklist along with the extracted data and critical appraisal. I have attached three different examples of extractions and appraisals which follow the SURE checklist approach and its corresponding full text reviews.”

As part of the training, we will trial automating extractions and appraisals for two additional reviews, originally manually extracted and appraised; this time requesting Copilot 365 to extract the data and critically appraise each review individually. This will allow us to fine-tune the quality of outputs generated by Copilot. The prompt will include the following:

“Appraise and extract data from the attached PDF labelled R005 for each heading on the SURE checklist attached. Use a narrative approach.”

Should it be required, we will direct Copilot 365 to adopt appropriate formats as we advance, ensuring consistency with the manual screening format and language. For example, if needed, we will guide Copilot to extract data in a narrative style for specific fields (e.g. background) and/or highlight key data that was initially overlooked by Copilot 365. Therefore, we are anticipating a total of three rounds of training to fine tune the LLM and ensure the desired output is generated.

Once training is completed, we will re-prompt Copilot 365 to extract data and critically appraise full text reviews of the remaining reviews:

“Appraise and extract data from the attached PDF labelled R005 for each heading on the SURE checklist attached. Use a narrative approach.”

Automated data extraction and critical appraisal will be performed on 20% of the reviews selected for inclusion, excluding those used for training purposes. The automated outputs and their corresponding prompts for each review will be logged on the Copilot platform and then exported into a Word document format. These results will subsequently be compared to the data extractions and appraisals carried out manually.

To evaluate time savings, we will document the duration required for both automated and manual extraction and appraisal processes for 20% of the identical set of reviews. We will log the total time taken in the unit of hours by two reviewers to manually extract data and critically appraise a single review—which includes the time one reviewer spends extracting data and appraising the review from start to finish, plus the time the second reviewer takes to verify the extraction for accuracy and completeness and review the appraisal judgments. The reviewers participating in these processes have experience in data extraction and critical appraisal of eye health reviews, including cataracts.

The reviewer automating these processes using Copilot will record the duration needed to extract and critically appraise the reviews until Copilot produces the desired output. This will encompass the training time required for Copilot 365 up to the point of generating the desired output (Fig. 3). As time taken to extract and appraise reviews.

### Data analysis

#### Title and abstract screening

We will calculate the proportion of relevant references prioritised by PS at 20% and 40% thresholds relative to the total number of relevant references identified manually across the entire sample based on title and abstract. We will use the following formula:

$$\mathrm{Proportion}\;\mathrm{of}\;\mathrm{relevant}\;\mathrm{citations}\;\mathrm{identified}\;\mathrm{by}\;\mathrm{PS}\;\left(20\%/40\%\right)=\left(\mathrm{number}\;\mathrm{of}\;\mathrm{relevant}\;\mathrm{references}\;\mathrm{found}\;\mathrm{by}\;\mathrm{PS}\;\mathrm{at}\;20\%/40\%\right)/\mathrm{total}\;\mathrm{number}\;\mathrm{of}\;\mathrm{relevant}\;\mathrm{references}\;\mathrm{manually}\;\mathrm{identified}\;\mathrm{at}\;100\%)\times100$$

To calculate the total number of relevant citations not identified by PS at 20% and 40% we will use the following formula:

$$\mathrm{Number}\;\mathrm{of}\;\mathrm{relevant}\;\mathrm{citations}\;\mathrm{not}\;\mathrm{identified}\;\mathrm{by}\;\mathrm{PS}\;\left(20\%/40\%\right)=\mathrm{Total}\;\mathrm{number}\;\mathrm{of}\;\mathrm{relevant}\;\mathrm{references}\;\mathrm{manually}\;\mathrm{identified}\;\mathrm{at}\;100\%-\mathrm{total}\;\mathrm{number}\;\mathrm{of}\;\mathrm{citations}\;\mathrm{of}\;\mathrm{relevant}\;\mathrm{citations}\;\mathrm{identified}\;\mathrm{by}\;\mathrm{PS}\;\left(20\%/40\%\right)$$

All screening decisions will be recorded automatically by ER, providing a comprehensive audit trail. We will generate reports on the screening process, which will be included in the final review documentation. We will evaluate the quality and comprehensiveness of the report generated on the screening process by ensuring all necessary details for the final review documentation [27].

### Full text screening

#### Accuracy

The accuracy of Copilot 365 in assessing the eligibility of reviews through full text screening will be evaluated by comparing the inclusion and exclusion decisions made by Copilot 365 against those made by human reviewers. This metric will gauge the model’s effectiveness in accurately predicting outcomes of full-text reviews. To accomplish this, we will.

1. Prepare the data: collect the decisions made by both Copilot 365 and human reviewers. Decisions will be presented as binary values − 1 for inclusion and 0 for exclusion.
2. Determine how many decisions made by Copilot 365 match the decisions made by human reviewers.
3. Calculate accuracy:
  $$\mathrm{Accuracy}\;\mathrm{percentage}=\left(\mathrm{number}\;\mathrm{of}\;\mathrm{correct}\;\mathrm{decision}\;\mathrm{by}\;\mathrm{Copilot}/\mathrm{total}\;\mathrm{number}\;\mathrm{of}\;\mathrm{decisons}\right)\times100$$

The literature does not define definitive thresholds for evaluating the accuracy of AI models; therefore, the percentage derived from the calculations will indicate how often Copilot 365 is correct.

#### Consistency

To assess the consistency of decisions between Copilot 365 and human reviewers, we will measure the inter-rater reliability (Table 4):

**Table 4:** Example of contingency table

Example	Human: include (1)	Human: exclude (0)
Copilot: include (1)	a (both include)	b (AI include, human exclude)
Copilot: exclude (0)	c (AI exclude, human include)	d (both exclude)

1. Collect data
  - Gather the inclusion/exclusion decisions made by both Copilot 365 and the human reviewers for each review. Represent these decisions in a binary format (e.g. 1 for include, 0 for exclude).
  - Create a contingency table: constructing a 2 × 2 table showing the counts of each possible decision pair (example below).
2. Calculate observed agreement (${\mathrm P\_}0$)
  $$P_{\mathit0}=\frac{\alpha+d}{\alpha+b+c+d}$$
3. Calculate expected agreement (P_e)
  $$P_e=\left(\frac{\left(\alpha+b\right)\cdot\left(\alpha+c\right)}{\left(\alpha+b+c+d\right)^2}\right)\;+\:\left(\frac{\left(c+d\right)\cdot\left(b+d\right)}{\left(\alpha+b+c+d\right)^2}\right)$$
4. Calculate Cohen’s Kappa
  $$\mathrm K=\frac{{\mathrm P}_0-{\mathrm P}_{\mathrm e}}{1-{\mathrm P}_{\mathrm e}}$$

Results from Kappa test will be interpreted as follows:

- 0—no agreement
- 0.10 to 0.20—slight agreement
- 0.21–0.40—fair agreement
- 0.41 to 0.60—moderate agreement
- 0.61 to 0.80—substantial agreement
- 0.81 to 1.00—almost perfect agreement

### Data extraction and critical appraisal

#### Accuracy and completeness

To assess the accuracy and completeness of automated data extraction and appraisal processes, a reviewer will manually inspect 20% of these outputs. We will measure the accuracy and completeness by calculating the proportion of correctly extracted data and appraisal judgments performed by Copilot 365. In this evaluation, we will verify if critical data outlined in the SURE checklist (such as inclusion criteria under the methodology section) were accurately extracted by Copilot 365. The methodology will include.

1. Identifying the total number of data points – determining the total number of data extraction points that need to be verified based on the SURE checklist.
2. Counting the correctly extracted data points: After manual verification, count the number of data points that were correctly extracted by the automation tool.
3. Calculating the accuracy percentage:
  i. Accuracy percentage = (number of correctly extracted data points/total number of data points) × 100
4. This process will be repeated for critical appraisals.

#### Time savings

To measure time savings by using Copilot 365, we will compare the time taken in the unit of hours for manual extraction and appraisal versus the time taken using Copilot 365. To calculate the time saved, we will subtract the time taken by Copilot 365 from the time taken manually.

Time Savings = (Time taken by human reviewers—Time taken by Copilot 365/Time taken by human reviewers) × 100.

#### Consistency

To understand how often Copilot 365 and the human reviewer agree or disagree on their assessments (consistency), we will calculate Cohen’s Kappa. This metric will measure the inter-rater reliability between Copilot 365 and the human reviewer for 20% of the assessments (Table 5). To calculate Cohen’s Kappa, we will.

**Table 5:** Example of contingency table

	Reviewer 2: yes	Reviewer 2: no	Total
Reviewer 1: yes	8	2	10
Reviewer 1: no	1	9	10
	9	11	20

*Reviewer 1* reflects Copilot assessments, *Reviewer 2* reflect human reviewer’s assessment

1. Create a contingency table.
  - List the categories that the human reviewer and Copilot 365 can assign: yes, no
  - Count the number of times each combination of ratings occurs
2. Calculate observed agreement (P0):
  $$Po=Number\;of\;\frac{Number\;of\;agreements}{Total\;number\;of\;assessments}$$
3. Calculate expected agreement (Pe):
  Pe = ∑(proportion of reviewer’s 1 rating for a category x proportion of reviewer’s 2 ratings for the same category)
4. Calculate Cohen’s Kappa
  $$\mathrm K=\frac{P_o-P_e}{1-P_e}$$

Results from Kappa test will be interpreted as follows: 0—no agreement.

- 0.10 to 0.20—slight agreement
- 0.21–0.40—fair agreement
- 0.41 to 0.60—moderate agreement
- 0.61 to 0.80—substantial agreement
- 0.81 to 1.00—almost perfect agreement

Combining these methods will provide an evaluation of Copilot 365’s effectiveness in automating data extraction and critical appraisal processes.

## Discussion

To the best of our knowledge, this is the first study to assess PS for titles and abstracts and employ Copilot 365 for automating full text screening, data extraction, and appraisal in updating an EGM. While AI can streamline systematic review processes, several limitations must be considered. The accuracy of AI tools is critical, and their performance must be rigorously compared to human reviewers to ensure reliability. This study will evaluate ER and Copilot 365 at different stages, acknowledging that human oversight may still be needed.

Effective use of AI tools requires user training and calibration. Training Copilot 365 using a set of manually screened and appraised reviews is essential to replicate human decisions accurately and reduce biased outputs, though it can be time-consuming and iterative. Accurate and unbiased training data is also key to ensure consistent answers/outputs from the LLM of interest [59]. Response variability can be influenced by factors such as a model’s contextual interpretation ability, diversity in training data, machine comprehension, language processing capabilities, and inherent biases. This variability may limit the reliability of findings and replicability of a review. The ability of LLMs, including Copilot 365, to generate coherent and fluent text may overshadow the factual inaccuracies included in the text. Furthermore, the quality of outputs generated by the LLMs is prompt-dependent, where guided prompts yield more reliable outputs compared to unguided prompting [38].

Another limitation of this study concerns the generalisability of the time savings performance measure by using Copilot 365 for data extraction and appraisal processes. We recognise that the time required to extract and critically appraise studies can vary significantly between reviewers, depending on their experience and the topic area. As a result, the observed time savings may not be generalisable. In this study, both reviewers involved in the extraction and appraisal of reviews have prior experience with earlier updates of eye health-related EGMs, including those focused on cataracts.

Although research demonstrates the potential of ER in reducing overall workload and the manual screening burden, the ideal manual screening threshold remains unclear, particularly for smaller reports where the number of citations to screen is small (approx. 300). Despite the anticipated challenges of using ER due to the small sample size of citations during the cataract EGM update, this study aims to contribute towards the evidence base by providing further insights into the accuracy of ER in screening small samples of citations. This study will help clarify the effectiveness of ER in such contexts and potentially guide future applications of automated tools in evidence synthesis for smaller datasets.

In evaluating Copilot 365, which shares similar features with other AI tools (e.g. ChatGPT, Gemini) we will gain valuable insights into the broader applicability and limitations of these types of AI tools in evidence synthesis. Copilot 365’s performance in tasks like full-text screening, data extraction, and critical appraisal can serve as a proxy for understanding how other AI tools might perform in similar contexts. This evaluation will help identify common strengths, such as increased efficiency and accuracy, as well as potential weaknesses, such as areas where human oversight remains crucial. Consequently, the results of this study will be relevant not only to Copilot 365 but also to other AI applications in the field, providing a foundation for their integration into systematic review processes and evidence synthesis workflows. This broader relevance underscores the potential for AI tools to transform evidence-based research by streamlining complex tasks and change how is research is conducted. Nevertheless, continuous improvement and ethical considerations are crucial in the development of AI tools for the automation of systematic review processes. As algorithms evolve, LLMs and other AI types will be expected to enhance accuracy and capabilities with potential for deeper and more meaningful exchanges between humans and AI [59].

## Supplementary Information

Additional file 1. Definition of terms used in the study protocol. Description of data: The document provides definitions for various terms used in the study protocol.

Additional file 2. SURE checklist.

## Data Availability

Not applicable.
