# Supplementary material for: Study protocol for evaluating automation of systematic review processes with EPPI-Reviewer and Copilot 365 in updating the cataract evidence gap map
Source: Syst Rev. 2026 Feb 17;15:95. doi: 10.1186/s13643-026-03101-4 (PMC13014901; doi:10.1186/s13643-026-03101-4)
Supplement: Supplementary file 1 — Additional file 1. Definition of terms used in the study protocol. Description of data: The document provides definitions for various terms used in the study protocol. [file 13643_2026_3101_MOESM1_ESM.docx]

**Additional file 1:** Definition of terms used in the study protocol

| **Terms** | **Definition** |
| --- | --- |
| Accuracy | Refers to how close the results and conclusions are to the true values or outcomes.(1, 2) |
| Artificial intelligence (AI) | Is a technology that allows computers and machines to simulate human intelligence in tasks like problem-solving, learning, and adaptation. (3, 4) |
| Automation | The use of technology to perform tasks with little human involvement, especially useful in evidence synthesis, where automation can simplify processes like screening and data extraction. (4) |
| Bias in research | A systematic error or deviation from the truth in research results or conclusions. It can happen at different stages of the research process and is a major concern in evidence synthesis when assessing the quality and reliability of included studies. (4) |
| Bidirectional Encoder Representations from Transformers (BERT) language model | A language model developed by Google that uses deep learning to understand and process text. In the context of evidence synthesis, BERT can be used to automate and enhance various tasks such as text classification, relation extraction, and named entity recognition.(5, 6) |
| Classification algorithms or classifiers | An algorithm that sorts data into different categories or classes. It learns from labelled data during training and then uses that knowledge to classify new, unseen data. (7) |
| Consistency (inter-reliability) | The degree to which different reviewers (human or AI) agree on the inclusion or exclusion of studies.(8) |
| Critical appraisal | Thorough and systematic evaluation of research to examine its trustworthiness, value, and relevance in a specific process, which is part of the evidence synthesis process.(4, 9) |
| Data extraction | The process of collecting important details from studies in a structured and consistent way using information from journal articles and reports. (10) |
| Deduplication | The process if eliminating duplicate records or data entries, especially in systematic reviewer where multiple databases might have the same studies, and these duplicates need to be found and removed.(4) |
| Deep Learning | A subset of machine learning that uses many layers of neural networks, known as deep neural networks, to mimic how the human brain makes decisions.(3) |
| Deep learning algorithms | Advanced machine learning techniques that analyse and learn from data, and automatically discover patterns and features in the data.(3) |
| Digital evidence synthesis tool (DEST) | Tools designed to automate tasks within the evidence synthesis process, such as searching, screening, and data extraction. They range from simple database management systems to sophisticated machine learning platforms. (4) |
| Efficiency | Refers to how effectively AI tools perform tasks compared to human performance, including measures of accuracy.(11) |
| Efficacy trials | Determine whether an intervention produces the expected results under ideal circumstances. (12) |
| Effectiveness trials | Measure the degree of beneficial effect under “real world” clinical settings. (12) |
| Generative AI | Uses deep learning models to create original content like long text, and high-quality images. In evidence synthesis generative AI can help automates tasks such as data extraction and summarising research findings. (3) |
| Heuristic cutoff point | Reviewers stop screening citations when a given number of irrelevant articles are seen in a row.(13) |
| Large language models | Tools trained in lots of data that can understand and create natural language and other content for many tasks. (14) |
| Machine learning | A subset of artificial intelligence that employs statistical techniques to enable computers to "learn" from data without being explicitly programmed.(3) |
| Machine learning/self-learning algorithms | A set of rules or processes used by an AI system to perform tasks, typically to uncover new data insights and patterns or predict output values from a given set of input variables. Algorithms enable machine learning (ML) systems to learn and improve.(15) |
| Narrative review | A review of the literature that provides comprehensive, descriptive summary of existing research on a topic.(16) |
| Natural language processing | A branch of computer science and artificial intelligence that leverages machine learning to help computers comprehend and interact with human language.(17) |
| Network meta-analysis | An approach to compare three or more treatments at the same time by using information from different studies. It combines both direct comparisons (from studies that directly compare treatments) and indirect comparisons (from studies that compare treatments through a common link).(18) |
| Precision | Proportion of correctly identified positives amongst all positives.(1) |
| Prioritisation screening | A process in systematic revies where studies are ranked or sorted by relevance using algorithms, which helps save time on manual screening. (4) |
| Recall (sensitivity) | Is the proportion of correctly identified positives amongst all real positive. (1) |
| Supervised learning | A basic machine learning method where algorithms are trained by humans using labelled data to predict outcomes or classify information.(3) |
| Support vector machine (SVM) | Supervised machine learning algorithm that classifies data by finings the best line or boundary that separates different classes in a multi-dimensional space.(19) |
| Systematic review | Gathers evidence that meets specific criteria to answer a research question. They aim to reduce bias by following clear, pre-planned methods outlined in a protocol. (20) |
| Systematic review toolbox | A collection of software tools and guidance documents designed to support the systematic review process. It helps researchers find resources for various tasks involved in evidence synthesis, such as screening, data extraction, and quality assessment. (21) |
| Systematic reviews of effectiveness trials | A research method where a comprehensive analysis is conducted by systematically identifying, evaluating, and summarising all available research studies (typically randomised controlled trials) that examine the "effectiveness" of a particular intervention or treatment in real-world clinical settings. (22) |
| Text mining | An automated process that helps identify and organise patterns in the text of single documents and across multiple documents. (23) |
| Unsupervised learning | A type of machine learning, where algorithms are used to identify patterns and relationships in data without any labelled examples.(3) |
| Validity | Refers to the accuracy and trustworthiness of the results and conclusions. It involved assessing whether the research methods and process used are free from errors or biases that could distort the findings.(24) |

**References**

1. O'Mara-Eves A, Thomas J, McNaught J, Miwa M, Ananiadou S. Using text mining for study identification in

systematic reviews: a systematic review of

current approaches. Syst Rev. 2015;4:22.

2. Ge L, Agrawal R, Singer M, Kannapiran P, De Castro Molina JA, Teow KL, et al. Leveraging artificial intelligence to enhance systematic reviews in health research: advanced tools and challenges. Syst Rev. 2024;13(1):269.

3. IBM. What is artificial intelligence (AI)? 2024 [Available from: <https://www.ibm.com/topics/artificial-intelligence>.

4. Scheelbeek P, Bond M, Callaghan M, Minx J, Hadida G, O’Mara-Eves A, et al. Digital Evidence Synthesis Tools for Climate & Health: Barriers and enablers for enhanced use of DESTs in the climate & health field. London, UK; 2024.

5. Edwards KM, Song B, Porciello J, Engelbert M, Huang M, F. A. AI-accelerated Design of Evidence Synthesis for Global Development. Computation and Language 2023.

6. Doneva SE, Qin S, Sick B, Ellendorff T, Goldman J-P, Schneider G, et al. Large language models to process, analyze, and synthesize biomedical texts: a scoping review. Discover Artificial Intelligence. 2024;4(1).

7. Thomas J, McDonald S, Noel-Storr A, Shemilt I, Elliott J, Mavergames C, et al. Machine learning reduced workload with minimal risk of missing studies: development and evaluation of a randomized controlled trial classifier for Cochrane Reviews. J Clin Epidemiol. 2021;133:140-51.

8. Hanegraaf P, Wondimu A, Mosselman JJ, de Jong R, Abogunrin S, Queiros L, et al. Inter-reviewer reliability of human literature reviewing and implications for the introduction of machine-assisted systematic reviews: a mixed-methods review. BMJ Open. 2024;14(3):e076912.

9. CASP UK. What is critical appraisal? 2025 [Available from: <https://casp-uk.net/what-is-critical-appraisal/>.

10. Schmidt L, Finnerty Mutlu AN, Elmore R, Olorisade BK, Thomas J, Higgins JPT. Data extraction methods for systematic review (semi)automation: Update of a living systematic review. F1000Res. 2021;10:401.

11. Shcherbiak A, Habibnia H, Böhm R, Fiedler S. Evaluating science: A comparison of human and AI reviewers. Judgment and Decision Making. 2024;19.

12. Gartlehner G, Hansen RA, Nissman D, al. e. Criteria for Distinguishing Effectiveness From Efficacy Trials in Systematic Reviews. Rockville (MD): Agency for Healthcare Research and Quality (US); 2006 April

13. Callaghan MW, Muller-Hansen F. Statistical stopping criteria for automated screening in systematic reviews. Syst Rev. 2020;9(1):273.

14. IBM. What are large language models (LLMs)? : IBM; 2025 [Available from: <https://www.ibm.com/think/topics/large-language-models?mhsrc=ibmsearch_a&mhq=what%20are%20large%20language%20models>.

15. IBM. What is a machine learning algortihm? : IBM 2025 [Available from: <https://www.ibm.com/think/topics/machine-learning-algorithms>.

16. Jahan N, Naveed S, Zeshan M, Tahir MA. How to Conduct a Systematic Review: A Narrative Literature Review. Cureus. 2016;8(11):e864.

17. IBM. What is NLP (natural language processing)? : IBM; 2025 [Available from: <https://www.ibm.com/think/topics/natural-language-processing>.

18. Cochrane Collaboration. Cochrane Handbook for Systematic Reviews of Interventions, Chapter 11: Network meta-analysis: Cochrane 2025.

19. IBM. What are support vector machines (SVMs)? : IBM; 2025 [Available from: <https://www.ibm.com/think/topics/support-vector-machine>.

20. Cumpston M, Flemyng E, Thomas J, Higgins JPT, Deeks JJ, MJ C. Chapter I: Introduction. In: Higgins JPT TJ, Chandler J, Cumpston M, Li T, Page MJ, Welch VA, editor. Cochrane Handbook for Systematic Reviews of Interventions version 65: Cochrane 2024.

21. Johnson EE, O'Keefe H, Sutton A, Marshall C. The Systematic Review Toolbox: keeping up to date with tools to support evidence synthesis. Syst Rev. 2022;11(1):258.

22. Munn Z, Stern C, Aromataris E, Lockwood C, Jordan Z. What kind of systematic review should I conduct? A proposed typology and guidance for systematic reviewers in the medical and health sciences. BMC Med Res Methodol. 2018;18(1):5.

23. Shemilt I, Simon A, Hollands GJ, Marteau TM, Ogilvie D, O'Mara-Eves A, et al. Pinpointing needles in giant haystacks: use of text mining to reduce impractical screening workload in extremely large scoping reviews. Res Synth Methods. 2014;5(1):31-49.

24. Evidence E. Critical Appraisal of Study Validity 2025 [Available from: <https://environmentalevidence.org/information-for-authors/7-critical-appraisal-of-study-validity/>.
